# Vital Dye Reaction and Granule Localization in Periplasm of *Escherichia coli*

**DOI:** 10.1371/journal.pone.0038427

**Published:** 2012-06-04

**Authors:** Liyan Ping, Despoina A. I. Mavridou, Eldon Emberly, Martin Westermann, Stuart J. Ferguson

**Affiliations:** 1 Department of Bioorganic Chemistry, Max Planck Institute for Chemical Ecology, Jena, Germany; 2 Department of Biochemistry, University of Oxford, Oxford, United Kingdom; 3 Physics Department, Simon Fraser University, Burnaby, British Columbia, Canada; 4 Centre of Electron microscopy, The University Hospital, Friedrich Schiller University of Jena, Jena, Germany; Montana State University, United States of America

## Abstract

**Background:**

Tetrazolium salts are widely used in biology as indicators of metabolic activity – hence termed vital dyes – but their reduction site is still debated despite decades of intensive research. The prototype, 2,3,5- triphenyl tetrazolium chloride, which was first synthesized a century ago, often generates a single formazan granule at the old pole of *Escherichia coli* cells after reduction. So far, no explanation for their pole localization has been proposed.

**Method/Principal Findings:**

Here we provide evidence that the granules form in the periplasm of bacterial cells. A source of reducing power is deduced to be thiol groups destined to become disulfides, since deletion of *dsbA*, coding for thiol-oxidase, enhances the formation of reduced formazan. However, pervasive reduction did not result in a random distribution of formazan aggregates. In filamentous cells, large granules appear at regular intervals of about four normal cell-lengths, consistent with a diffusion-to-capture model. Computer simulations of a minimal biophysical model showed that the pole localization of granules is a spontaneous process, i.e. small granules in a normal size bacterium have lower energy at the poles. This biased their diffusion to the poles. They kept growing there and eventually became fixed.

**Conclusions:**

We observed that formazan granules formed in the periplasm after reduction of tetrazolium, which calls for re-evaluation of previous studies using cell-free systems that liberate inaccessible intracellular reductant and potentially generate artifacts. The localization of formazan granules in *E. coli* cells can now be understood. In living bacteria, the seeds formed at or migrated to the new pole would become visible only when that new pole already became an old pole, because of the relatively slow growth rate of granules relative to cell division.

## Introduction

Tetrazolium salts have been widely used in assays that measure cell proliferation. Some of them form water-soluble formazans after reduction, while others form insoluble granules [1]. Their applications in eukaryotic systems are exemplified by the large-scale drug-screening programs hosted by the National Institutes of Health. These dyes also have applications in microbiology, e.g., 5-cyano-2,3-ditolyl tetrazolium chloride (CTC) was used to enumerate metabolically active bacteria in environmental samples [1], [2], [3] as well as in stationary phase cultures [4]. The rationale behind these applications is that in a cell culture or living tissue, dye reduction is proportional to cell metabolic activity. However, not all living cells in a culture show the reduction activity, and reduction is significantly influenced by factors such as the type of dye, the pH of the medium, and cell line in use [1], [3], [5].

Understanding the reduction mechanism is therefore critical for developing the next generation of dyes as well as for evaluating current results. As vital dyes, tetrazolium salts are known to accept hydrogen from the respiratory oxidation system, and is often assumed to be reduced intracellularly [1], [6]. However, the use of cell-free systems to identify reduction sites has been only partially successful in eukaryotic systems [1]. Accumulating evidence indicates that *in vivo* reduction pathways are very different from those in *in vitro* systems, e.g. inhibitors of the succinate:ubiquinone oxidoreductase pathway can completely block CTC reduction in membrane vesicles but have no effect on intact *Escherichia coli* cells [7].

2,3,5- triphenyl tetrazolium chloride (TTC) was synthesized a century ago and is the prototype of all tetrazolium dyes [1]. Lederberg applied it to *E. coli* in 1948, and observed large granules at one of the two cell poles [8]. Berg and Turner used these granules as the pole marker to study cell orientation in swimming bacteria [9]. One of the two bacterial cell poles is derived from the septum (new pole), while the other is inherited from the parental generation (old pole) [10]. We have shown previously that the granules were often located at the old pole [11]. Spontaneous localization of self-aggregating proteins in bacteria has been described for membrane receptors [10], [12], cytoplasmic proteins such as DivIVA [13], [14] and PopZ [15], [16], and misfolded proteins [17]. A variety of mechanisms have been proposed to explain the patterns from diffusion to capture [18], to membrane curvature [19], [20] and to nucleoid occlusion [17], [21]. Here, we provide for the first time experimental and theoretical evidence that TTC is reduced in the periplasm and that aggregation of small molecules, such as the reduced formazan, at the cell poles is a spontaneous process.

## Results and Discussion

### Granules Formed in the Periplasm of *E. coli* Cells

**Figure 1:**
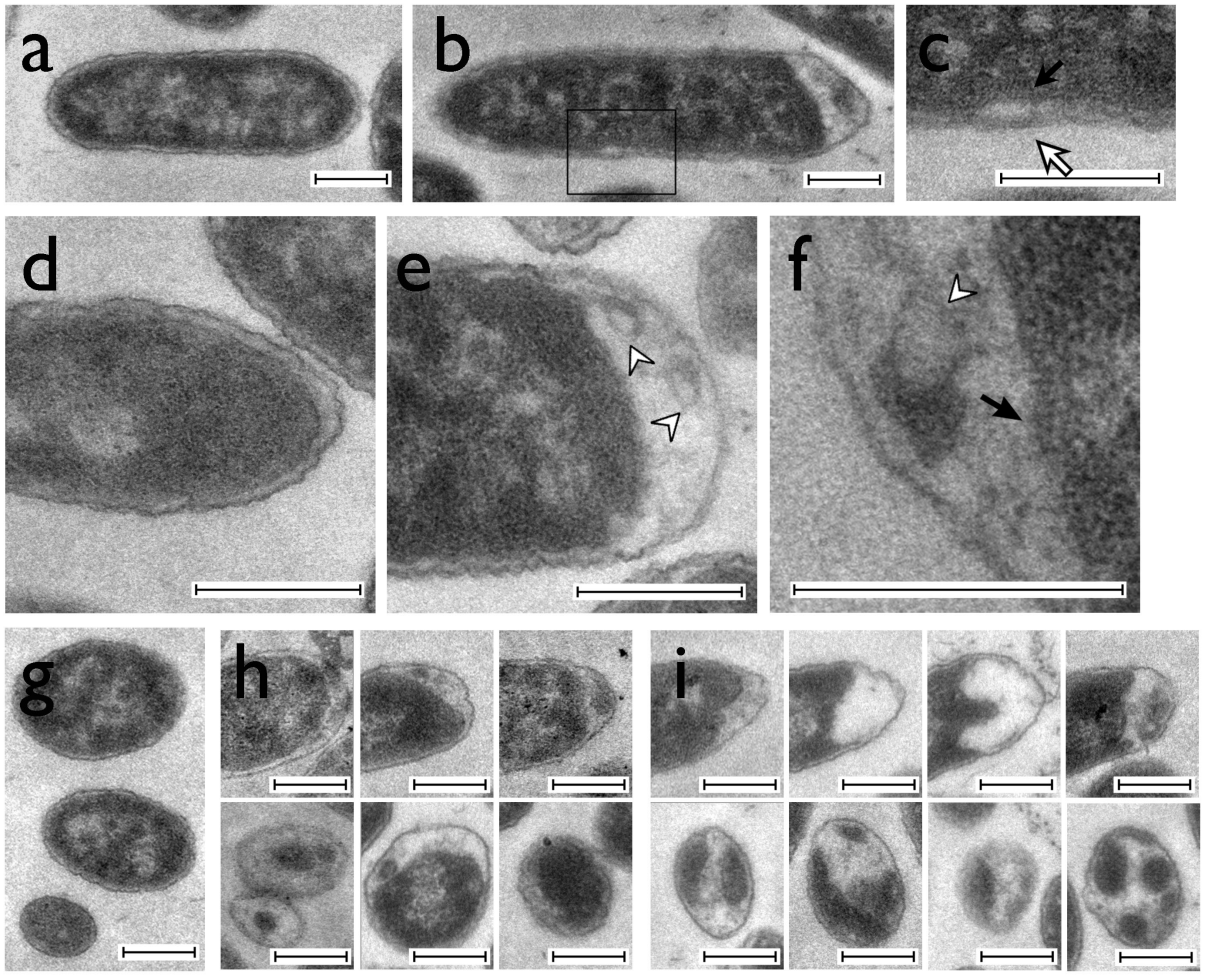
Transmission electron microscopy images of *E. coli* cells containing formazan granules. Scale bars equal 400 nm. A, Longitudinal section of a control cell growing in medium without tetrazolium. B, Longitudinal section of a cell growing in the presence of TTC. The formazan granule is located at the right pole of the cell. C, Expanded view of the boxed area in panel. The cytoplasmic membrane is indicated by a closed arrow. B. The small lateral granule is indicated by an open arrow. D, Expanded view of the pole area of the control cell. E, Expanded view of the pole of a cell growing in TTC. The concentric contour lines on the cutting surface of the granule are indicated by open arrowheads. F, A further expanded picture of a cell pole, showing the concentric contour lines (open arrowhead) and the cytoplasmic membrane (closed arrow). G, An ultrathin section in which three control cells were cut at different positions, the bottom one being at the tip of the pole. H, Ultrathin sections of poles of cells growing in a medium containing tetrazolium for 12 h. (upper panels: longitudinal sections; lower panels: transverse sections). I, Sections of the poles of cells growing in the presence of tetrazolium for 18 h (upper panels: longitudinal sections; lower panels: transverse sections).

Formazan granules are often found at the old pole of *E. coli* cells. We have already shown that the granules refracted fluorescence from GFP fused to the membrane serine receptor Tsr, making the pole look dimmer than non-treated cells [11], suggesting an out-of-membrane localization. When *E. coli* cells were fractionated as described by Ausubel *et al.* [22] with the EDTA (ethylenediaminetetraacetate) treatment step omitted, after digestion of the outer cell membrane and release of the periplasm; the insoluble granules were obviously floating in the periplasmic fraction (Data not shown). Transmission electron microscopy (TEM) performed on strain LMG194 that was employed for studying granules localization before [11] confirmed that large formazan granules were mainly formed in the periplasm and localized at the poles of the cell (Fig. 1). Using TEM we were also able to detect randomly distributed tiny lateral granules (Fig. 1C), which would not be observed by light microscopy. It is worth noting that the formazan granules are electron lucent due to their chemical composition, and even slightly lighter than the areas of the periplasm devoid of granules on TEM images. We only consider that a tiny granule is present when we observe a particle with low uniform electron density that is at least twice as thick as the width of the periplasm and is clearly bound by the electron dense cytoplasmic membrane and cell wall. In some cases, concentric contour lines on the cutting surface of the granule were also visible (Fig. 1E), suggesting that formazan had precipitated layer by layer. At many locations on the TEM images, the triple-layered cytoplasmic membrane was clearly visible (Fig. 1F), demonstrating that neither the granule formation nor the sample preparation caused any mechanical damage or dissolution of the cytoplasmic membrane [23]. Granules that were not large enough to be visualized under the light microscope were cap-shaped (Fig. 1H). The centers of these granules were often located on one side of the pole cap. The large granules visible under the light microscope were round on the tip side and exhibited random extrusions on the side facing the cytoplasmic membrane (Fig. 1I).

A great number of enzymatic and non-enzymatic biological reductants are known to reduce tetrazolium dyes *in vitro* [1]. However, most of these molecules are inaccessible to the vital dyes in a living cell. To our knowledge, the experiments presented here provide the first evidence that reduced formazan forms granules in the bacterial periplasm (Fig. 1). The turgor pressure from the cytoplasm probably plays an important role in shaping the granules: they are cap-shaped at the cell pole when they are small but compress the cytoplasmic membrane with random extrusions when they are large. In long filamentous cells, the larger volume of cytoplasm allows for a more extensive membrane deformation; the granules at the pole can grow much larger and remain round (Fig. S1).

The localization of formazan granules in living eukaryotic cells has been shown to depend on the permeability of cellular membranes to the dyes under investigation. 3-(4,5-dimethylthiazol-2-yl)-2,5-diphenyltetrazolium bromide (MTT) and TTC can readily cross some eukaryotic plasma membranes [24], [25], but not the mitochondrial inner membrane [26]. CTC, however, cannot efficiently pass through the eukaryotic plasma membrane [24]. The majority of MTT-granules were formed adjacent to the mitochondrial inner membrane [24], [27], and most of the CTC-granules co-localized with the plasma membrane [24], [28]. It could be said that the bacterial cytoplasmic membrane plays a dual role, that of the eukaryotic plasma membrane and of the mitochondrial inner membrane. Therefore, the accumulation of TTC-granules in the bacterial periplasm is consistent with the localization of formazan granules in eukaryotic cells.

### TTC Reduction Pathway

To elucidate the pathway of TTC reduction in the bacterial periplasm, null mutants of proteins from two pathways that play a major role in determining the redox state of this compartment were analyzed (Table S1). Oxidative protein folding in the periplasm of Gram-negative bacteria relies on the concerted action of five proteins comprising the Disulfide bond formation (Dsb) system that functions through a series of thiol-disulfide exchange reactions. The thiol-oxidase DsbA is responsible for the formation of disulfide bonds for proteins in the periplasm [29]. Its active oxidized form is maintained by the transmembrane protein DsbB, which is oxidized by ubiquinone [30]. Wrongly-formed disulfide bonds are corrected by DsbC [31], which acquires its reducing power from the transmembrane protein DsbD [32]. DsbD also reduces DsbG, which has a role in protecting single cysteines from oxidation to sulfenic acid on folded periplasmic proteins [33]. Another source of reducing power in the bacterial periplasm is *c*-type cytochrome maturation (Ccm) system, specifically the CcmG component which is a periplasmic thioredoxin-like protein [34].

**Figure 2:**
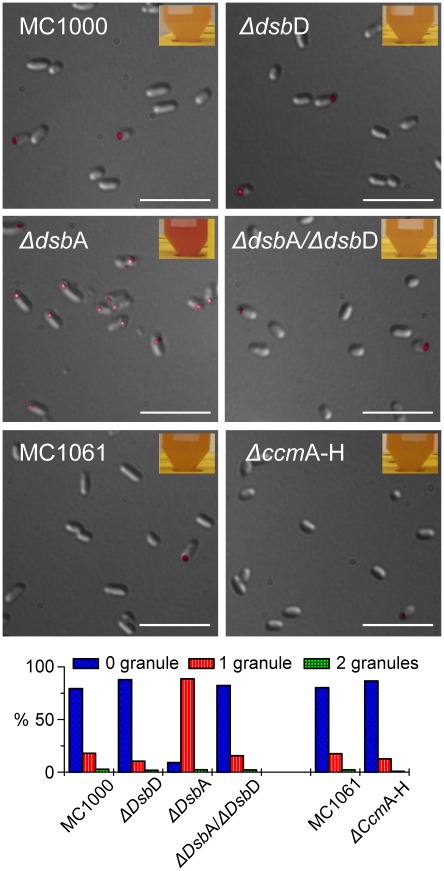
Formazan granule formation in different *E. coli* mutants. Scale bars equal 20 µm. Granules were false-colored in dark magenta. The top two lines show the parental strain (MC1000) and its derivatives: the *dsb*D deletion, the *dsb*A deletion, and the *dsbA*/*dsbD* double mutant strains. The third line shows the parental strain (MC1061) and the Ccm null mutant derived from it. Photos of the corresponding cell cultures after growth in the presence of TTC are shown at the top-right corner of each DIC image. The percentage of cells containing different numbers of granules for each strain is compared in the histogram at the bottom of the figure.

We tested mutants in the Dsb pathway that blocked either the reducing power inlet (*Δdsb*D) or the oxidizing power outlet (*Δdsb*A). The accumulation of formazan granules in *Δdsb*D strain showed no difference from the parental strain (Fig. 2). However, the reduction of TTC was significantly enhanced in the *Δdsb*A strain under identical growth conditions. The extensive accumulation of granules in the *dsbA* deletion strain was obvious even by simply examining the intense red color of the cell culture (inset). In the *dsbA* and *dsbD* double deletion strain, the formation of granules was at the same levels as in the parental strain. When the whole *ccm* operon was deleted, no change in granule formation was observed (Fig. 2).

This result indicates that the reduction of TTC in bacterial cells is rather non-specific. The redox potential in the periplasmic space of *E. coli* cell is −165 mV [35], while the reduction potential of TTC at pH 6.72 is −80 mV [36]. Therefore, many components of the bacterial periplasmic redox system can reduce TTC at a physiological pH. The lack of the main oxidative protein DsbA, nevertheless, resulted in a more reducing periplasm, which can explain the enhanced TTC reduction. Very aerobic conditions could compensate for the lack of DsbA; however, in the present work the cells grew microaerobically. When the electron flow from the cytoplasm was blocked through deleting DsbD, the TTC reduction in the *Δdsb*A strain returned to a level similar to that of wild-type cells. The antagonistic effect of a *dsbD* deletion further supported the assumption of non-specific reduction.

The electrons transferred into the periplasm by DsbD and other transporters eventually go back to terminal oxidases and oxygen [34], [35]. We hereby tested the influence of the essential isoprenoid quinone components of the respiratory electron transport chain on TTC reduction. The reduction activity of the ubiquinone-deficient strain AN385 under anoxic condition and of the menaquinone-deficient strain AN386 under oxic condition were not significantly different from the parent strain AN387 under same conditions (Table S1). However, in strain AN384, which is deficient on both ubiquinone and metaquine, TTC could not be reduced. This result is consistent with the general assumption that the reducing power is derived from cellular respiration and also the observation on *Lactococcus lactis* cells, which perform lactic acid fermentation and contain only menaquinone [37], deleting menaquinone biosynthesis genes in *L. lactis* completely blocks the reduction of tetrazolium violet [38]. Nevertheless, the existence of a major terminal reductase for TTC that is DsbA-dependent could still be considered. However, the tested potential periplasmic oxidoreductases, including DsbD, CcmG, and CcmH (Our Ccm-null strain includes deletion of CcmG and of the *N*-terminal domain of CcmH) did not implicate any of these proteins.

### Diffusion of Formazan in Periplasm

**Figure 3:**
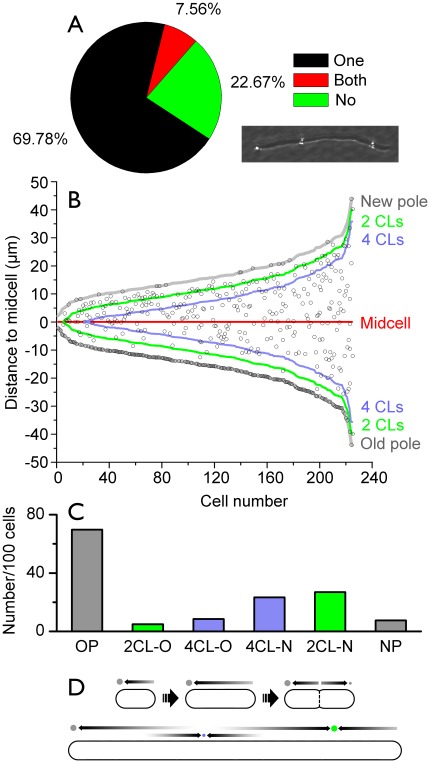
Localization of formazan granules on filamentous *E. coli* cells. A, Percentage of filaments with different numbers of granules on the cell poles (n = 225). The inset shows a typical cell with three granules. They are bright spots in the dark-field image. B, The distribution of granules (open dots) on the filaments relative to the geometrical midpoint of cells. The green and blue lines mark the position of 2 and 4 cell-lengths (CLs) from the cell poles, respectively. C, The number of granules per 100 cells with the same color scheme as in B: OP, old pole; 2CL-O, the region within 2 CLs of the old pole; 4CL-O, the region between 2 CLsand 4 CLs of the old pole; 4CL-N, the region between 2 CLs and 4 CLs of the new pole; 2CL-N, the region within 2 CLs of the new pole; NP, new pole. D, A diffusion to capture model of granule formation. The old poles of the cells are depicted on the left-hand side. Arrows represent the diffusion of reduced formazan. The three cells shown on top are newborn, fully elongated, and dividing cells. The broken line represents a septum. A filamentous cell of 8 CLs is shown below.

TTC is reduced non-specifically in the periplasm, but most cells contain only one large granule at the pole [11]. To further understand the mechanism of granule localization, we generated filamentous cells using cephalexin [39] (Fig. 3). The average length of filaments under investigation was 19.7 µm, which equals 9.4 normal cell-lengths (CLs). Here CL was defined as the average length of newborn cells in a normal population (the value of the lower quartile of a random sample was used for approximation (n = 160). Like normal cells, 70% of the filaments contained a large granule at the old pole (Fig. 3A). Lateral granules on filaments were regularly spaced (Fig. 3 B). The average distance between two formazan granules and between the granules and poles was 6.7 CLs. The most granule-rich region on the lateral periplasm was about two CLs away from the new pole. Very few granules were found at the same distance from the old pole (Fig. 3C). However, the granule number significantly increased at 4 CLs from the old pole.

The distribution of granules on filaments can be explained by a diffusion to capture scheme. In such a mechanism, a large granule acts as an sink for all nearby diffusing formazan molecules, that sets up a formazan concentration gradient which is characterized by a diffusion length that depends on the diffusion coefficient of formazan in the periplasm and on the rate at which it is being added to the periplasmic space. The periplasmic space in cephalexin-induced filamentous cells is continuous [40]. Slowly aggregating but quickly diffusing substances such as the insoluble formazan formed regularly spaced granules, because existing granules capture all the formazan molecules within the diffusion length and suppress the formation and growth of others nearby (Fig. 3D). On TEM images of normal sized cells, small randomly distributed lateral granules were indeed sometimes observed (Fig. 1C). This experiment indicates that pre-existing granules could create a formazan gradient up to 3–4 CLs long. The distance between two sinks should be doubled (7 CLs) as experimentally determined. A normal cell is much shorter than the formazan diffusible distance, therefore only one large granule could be formed (Fig. 3D).

### Simulation of Granule Localization and Experimental Verification

The diffusion to capture theory, however, could not explain the polar localization of granules. A minimal biophysical model of formazan aggregation and diffusion in the periplasmic space was therefore constructed. The inner and outer membranes were modeled as rigid hard walls, defining a confining shell in which formazan molecules can diffuse and aggregate (Fig. S2). Molecules were modeled as spherical particles with a diameter *σ* were added uniformly within the periplasmic space at a constant rate. An isotropic attractive interaction between particles governed by a Lennard-Jones potential with a well depth *ε* that allows them to grow as aggregates was given by
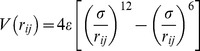
where *r_ij_* is the distance between particles *i* and *j.* We explored granule formation at various rate of addition, strength of interaction and size of the periplasmic space.

**Figure 4:**
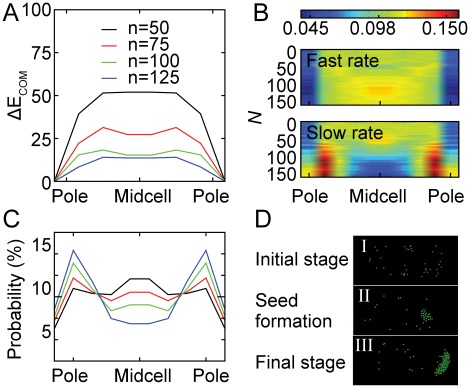
Computer simulation of the formazan diffusion in cell periplasm. A, Average free energy of a granule as a function of its center of mass (ΔE_COM_) along the longitudinal cell axis. The energy of the granule at the poles was taken as zero. The curves correspond to different numbers of particles. B, Frequency of granule location as a function of position on the cell and aggregate size (*N*). The heat map of particles added at fast rate (*N_add_*  = 10,000 MC steps) is shown above. The map of granules added at slow rate (*N_add_*  = 50,000 MC steps) is shown below. The top color scheme shows the frequency value. C, Probability of granule localizing to different positions on cell at slow addition rate. Line colors are the same as in A. D, Snapshots of three critical steps of aggregate formation in a simulation movie. I, initial randomly diffusing particles; II, a seed forms stochastically; III, diffusion to and growth at the energetically favored cell pole.

If granule formation and positioning in the periplasm is at equilibrium, we would expect the distribution of granules to be consistent with the free energy of a granule at a given position. Positions corresponding to lower energy would be more frequently occupied. We therefore looked at the total energy, *i.e.* the interaction energy between particles, for different numbers of formazan particles and granule positions in the periplasm. Simulations of the model revealed that small spherical granules have a much lower energy at the pole compared to midcell (Fig. 4A). We found that the average number of molecules in a granule at the pole is larger than that at midcell for equal concentration of molecules in periplasm. We attribute this to a smaller off-rate at the pole that particles dissociate from the granule due to spatial constrains compared to midcell. For larger granules that achieved disk-like shapes, the energy difference between the pole and midcell became less significant (Fig. 4 A). Given the small difference in energy between large midcell and pole granules, if the system could come to equilibrium, the spatial distribution of granules would be more uniform than experimentally observed, where granules localize at the poles in ∼70% of cells (For example see Fig. S5).

Since the observed localization frequency is different from what would be expected from equilibrium arguments, we explored the effect on localization due to the rate of addition of molecules. Regardless of addition rate, a seed was likely to form anywhere within the periplasmic space (Fig. 4B). If molecules were added at a rate faster than that required for them to diffuse to the lower energy position at the pole, the seeds could be trapped in the local energy minimum at midcell. If molecules were added slowly enough so that the small aggregates had enough time to migrate to the pole, then they formed a large aggregate there and reached a quasi-steady state. The experimentally observed ratio of pole-granule containing cells was obtained when the addition rate was slower than the typical time of a seed to diffuse from midcell to the pole (Fig. 4C). The simulation also revealed that the peak of large aggregates was slightly off pole, due to the increased entropy associated with that location, in good agreement with the experimental data (Fig. 4D and Fig. 1).

We also explored how granule formation depends on the width of the periplasmic space and the strength of molecular interaction. In both very wide and very narrow periplasms, the distribution of granules was far more uniform (Fig. S3). These results make intuitive sense as in wider periplasms, the geometrical constraint on granule growth and distribution was reduced; while in narrower periplasms, seeds growing in essentially 2D directions must reach a much larger size to overcome the energy barrier. When we varied the interaction energy between particles, we found that seeds formed at higher concentrations with lower interaction energy. They still tended to migrate to the pole, though the quasi-steady location was more variable; with higher interaction energy, seeds formed more frequently, and there were more granules forming at non-pole locations (Fig. S3).

The periplasm at the cell poles is a favored place for localization. It has been demonstrated that GFP expressed in the periplasm relocates to the pole when subjected to a mild osmotic shock [41]. The osmotic shock provides a mechanical force to propel GFP proteins into the energetically more favorable location. GFP is huge (4.5 nm×3.5 nm×3.5 nm) compared to the narrow periplasmic space (about 10 nm). The periplasm is also full of biological ‘gel’ [23], [42] and the diffusion coefficient of GFP in the periplasm has been measured as 2.6 µm^2^/s, much lower than in cytoplasm [40]. A formazan molecule, that is about 155 times smaller than GFP and its small aggregates would diffuse more readily towards the pole.

The diffusion constant of small molecules in water at room temperature has been estimated to be ∼1,000 µm^2^/s [43]. Without additional obstacle, a formazan molecule can diffuse 1 µm in 0.5 ms. The diffusion coefficient of a particle is reciprocally proportional to the square root of its size according to Stokes–Einstein equation [43]. The diffusion coefficient of a formazan molecule in periplasm, according to the GFP value, can be estimated as ∼372 µm^2^/s. A formazan molecule would thus diffuse 1 µm in ∼1.3 ms. Considering the potentially great interaction between GFP and the cell membranes, we expect the diffusion rate of formazan molecules *de facto* to be faster.

Our computer simulation indicated that the experimentally observed granule localization could be obtained only if the TTC reduction rate was slower than the time requires for a seed to defuse from midcell to the pole. It is worth noting that the TTC reduction in *E. coli* cells is slow. To observe enough granule-containing cells, bacteria were either cultivated in liquid culture at 23°C [9], [11], or on Petri dish [8]. Under fast growth conditions, the granule-containing cells were hard to analyze due to the dilution effect of cell division. To roughly estimate the reduction rate in periplasm, we measured six largest granule cutting surfaces on the ultrathin sections of cells growing for 2 h, 4 h, and 6 h in the presence of TTC (Fig. S4). The granule volume was estimated as a 10-µm-thick flat sheet, because membrane deformation did not occur up to 12-h incubation (Fig. 1). This result indicated that a granule needs ∼10 s to grow large enough to be trapped in the periplasm and the cell reduced one TTC molecule per 6 ms, much longer than the time needed for them to diffuse to the pole. The simulation also indicated that high reduction rate would enhance the non-pole localization of granules. We increased the reduction rate by adding more TTC into the medium (Fig. S5). As expected, the number of non-pole granules and the number of multiple-granule cells increased when more TTC was supplemented.

### Final Remarks

TTC is very likely reduced in periplasm non-specifically. The ubiquitous thioredoxin super-family proteins in *E. coli* periplasm might play a prominent role in vital dye reduction. This assumption is potentially applicable to intact eukaryotic cells. It has been observed that chemicals blocking sulfhydryl groups inhibited TTC reduction as efficiently as those decoupling respiration and phosphorylation in plant tissues [44]. The sulfhydryl groups and the formazan reduction has also been observed to co-localize [45]. In human neutrophils, only the activity of glutathione reductase, not the respiration oxidoreductases, shows a strong correlation with the reduction of nitroblue tetrazolium [46].

The bacterial periplasm provides a unique geometry for aggregating molecules to diffuse and to grow. The localization pattern would not be obtained in very wide or restrained spaces. A diffusion to capture model had been used to explain the regular spacing of lateral clusters of chemo-receptors along the cell body [12], [18] and this scenario is also applicable to small molecules, such as reduced formazan in periplasm. Using a minimal model, we showed that the periplasm at the cell poles is the most energetically favorable position for diffusing small molecules, and explained why the prominent granules often localize to the pole.

This model is potentially applicable to other self-aggregating small molecules entering the bacterial periplasm. Different molecules have different interaction energy, hence different aggregate localization pattern. The granule localization in eukaryotic cells would follow the same rule with a more sophisticated geographical boundary condition. It is also applicable to analogous systems, such as the chemoeffectors in chemotaxis. Chemoeffectors bind to the corresponding receptors or ligand-binding proteins, which would serve as a sink [47]. The formation of a maltose-binding-protein pole cap in *E. coli* upon maltose induction supports this assumption [48]. Interestingly, both clustering of receptors and migration of effectors are spontaneous processes favoring the cell poles. Whether these are simply biological spandrels or the manifestation of physical principles governing the evolution of rod-shaped bacteria would be an interesting question to address in the future.

## Materials and Methods

### Bacterial Strains and Growth Conditions

MC1000 [49] and MC1000*Δdsb*D [32] have been published previously. Strain MC1000*Δdsb*A and strain MC1000*Δdsb*A/*Δdsb*D were gifts from Prof. Linda Thöny-Meyer. Strain UT481 was a gift from Prof. Piet. A. J. de Boer. Strain AW405 was a gift from Prof. S. Parkinson. Strain AN384, AN385, AN386 and AN387 [50] were gifts from James A Imlay. LMG194 was obtained from Invitrogen. Bacteria were cultivated according to the published procedure [11] unless otherwise specified. In brief, a single colony was grown at 23°C and shaken at 160 rpm with aeration (doubling time: 147±17 min) to early stationary phase (OD_600_ = 1.1). 100 µl of this culture was used to inoculate 10 ml of fresh medium that was then incubated at the same temperature for 12 h with aeration. TTC (0.005% w/w), if needed, was added 0.5 h after inoculation.

### Transmission Electron Microscopy

Strain LMG194 was cultivated in LB medium with TTC for 12 h and 18 h. Cultures without TTC were used as controls. Cells were spun at 10,000 g for 60 s and resuspended in 1× phosphate buffered saline (PBS). The pellet was washed with PBS 3 times and fixed overnight with 4% glutaraldehyde. Cells were kept in PBS at 4°C before use. Samples were further fixed with 1% osmium tetroxide in sodium cacodylate buffer for 2 h and were dehydrated with ethanol in serially increased concentration. Cells were infiltrated with araldite resin and the resin was cured at 60°C for 48 h. Embedded samples were sectioned on a LKB 8800A Ultratome III (LKB Produkter AB, Bromma, Sweden). Ultra-thin sections were placed on Formvar-coated grids and contrasted with lead citrate for electron microscopy (EM 902; Zeiss, Oberkochen, Germany). To measure the growth rate of granules in periplasm, the cells of strain AW405 growing for 2 h, 4 h, and 6 h after addition of TTC were harvested and sectioned. Six largest cutting surfaces of granules were selected, and the areas were measured using ImageJ 1.37 V (National Institutes of Health, Bethesda, MD, USA). The volumes of the granules were approximated as a flat sheet of 10 nm thick.

### TTC Reduction in Different *E. coli* Strains

All mutant strains (Table S1) were grown in parallel with their parent strains for a given set of experiments. Samples used for microscopy were prepared as following: One ml of each cell culture was centrifuged at 5,600 g for 1 min, cell pellets were resuspended in 100 µl of growth medium, and then further diluted 5-fold with motility buffer (11.2 g l^−1^ K_2_HPO_4_, 4.8 g l^−1^ KH_2_PO_4_ and 0.029 g l^−1^ EDTA). One µl of the final suspension was immobilized on glass slides by 1% agarose gel and examined by Differential Interface Contrast (DIC) microscopy using a 250 × oil lens on a commercial Nikon TE2000/TIRF microscope. Strain AN384, AN385 and the parent strain AN387 was cultivated anaerobically in the presence of 1 mM 4-hydroxybenzoic acid in a sealed tube filled with argon, and assays were performed anaerobically with cells resuspended in fresh medium without 4-hydroxybenzoic acid. To further evaluate the influence of TTC reduction rate on granule localization, the cells of strain AW405 were cultivated in the presence of 0.005%, 0.05% and 0.1% TTC for 12 h, 10 h, and 8 h respectively. Five µl cell cultures were directly mounted on SuperFrost Ultra Plus adhesion slides (Thermo Scientific). Imaged was taken with an Axioskop microscope (100 × oil lens, Carl Zeiss). The granules that appeared as black spots were counted manually on randomly taken bright field images.

### Cell Filamentation Induced by Cephalexin

Strain LMG194 was grown in LB and was induced with 60 µg/ml cephalexin as described [51]. Cells mounted on SuperFrost Ultra Plus adhesion slides were imaged using an Axioskop microscope. The formazan granules appeared as bright spots on the dark-field images (Fig. 2A inset). The images were analyzed using ImageJ. Formazan granules within the cavity of the pole or immediately adjacent to the base of the pole were regarded as pole granules. On cells with zero or two pole granules, the pole farther away from the nearest lateral granule was assigned as the old pole.

### Computer Simulation of Granule Formation

The periplasm was modeled as a cylindrical shell of length, *L*, capped with two hemispheres of inner and outer radius, *R_I_* and *R_O_*, representing the radii of the inner and outer membranes respectively (Fig. S2). The system was modeled using a cell with an aspect ratio of (*L+*2*R_O_*)*/*2*R_O_  = 2*. The membrane boundaries were considered to be hard walls. For these simulations, *L*  = 2*R_O_*  = 8σ, and the outer membrane radius *R_O_*  = 4σ. The inner radius was chosen as *R_I_*  = 2.6σ in most simulations except when the influence of spatial constraint was considered. The energy of interaction between formazan particles was taken to be *ε*  =  n*k_B_T*, where n = 2 for most simulations except when the influence of interaction energy was evaluated.

The system was simulated using a Metropolis Monte Carlo (MC) method at constant temperature. In a single MC sweep, every particle was randomly moved with a maximum step size of *δr  = *0.1σ. Random moves were either accepted if the total energy was lowered, or rejected based on the Metropolis criterion. A given MC sweep through all particles can be interpreted as a single step in time, where all particles have had a chance to diffuse some distance *δr*. To construct the energy landscape of a granule as a function of its position in the cell, a fixed number of particles were added to the space and the location and energy of the granule was periodically sampled over a long equilibration run and the averages calculated. To build up the frequency of aggregate locations, 150 independent MC simulations starting from one particle to a final particle number, *N* were performed. The rate of addition of formazan to the periplasm was simulated by adding one particle randomly within the periplasm every *N_add_* MC sweeps. Thus by making *N_add_* larger or smaller, different rates of addition of formazan could be studied. The number of particles, the number of particles in the largest granule, the position of the center of mass of the largest granule, and the energy of the system was retrieved at fixed intervals in a simulation.

## Supporting Information

Figure S1 **Transmission electron microscopy images of a formazan granule in a filamentous cell of** ***E. coli*** **strain UT481.**
(DOC)

Figure S2 **Schematic of cell geometry used for simulation.**
(DOC)

Figure S3 **Heat map of aggregate sizes corresponding to different periplamic locations (horizontal axis) and different numbers of particles per cell (vertical axis).**
(DOC)

Figure S4 **Estimation of the growth rate of formazan granules in** ***E. coli*** **periplasm.**
(DOC)

Figure S5 **The localization of formazan granules in cells of strain AW405 growing in medium supplemented with different amount of TTC.**
(DOC)

Table S1 **Bacterial strains and their TTC reduction activities.**
(DOC)
