# Supplementary material for: Vital Dye Reaction and Granule Localization in Periplasm of Escherichia coli
Source: PLoS One. 2012 Jun 4;7(6):e38427. doi: 10.1371/journal.pone.0038427 (PMC3366950; doi:10.1371/journal.pone.0038427)
Supplement: Figure S1 — Transmission electron microscopy images of a formazan granule in a filamentous cell of E. coli strain UT481. (DOC) [file pone.0038427.s001.doc]

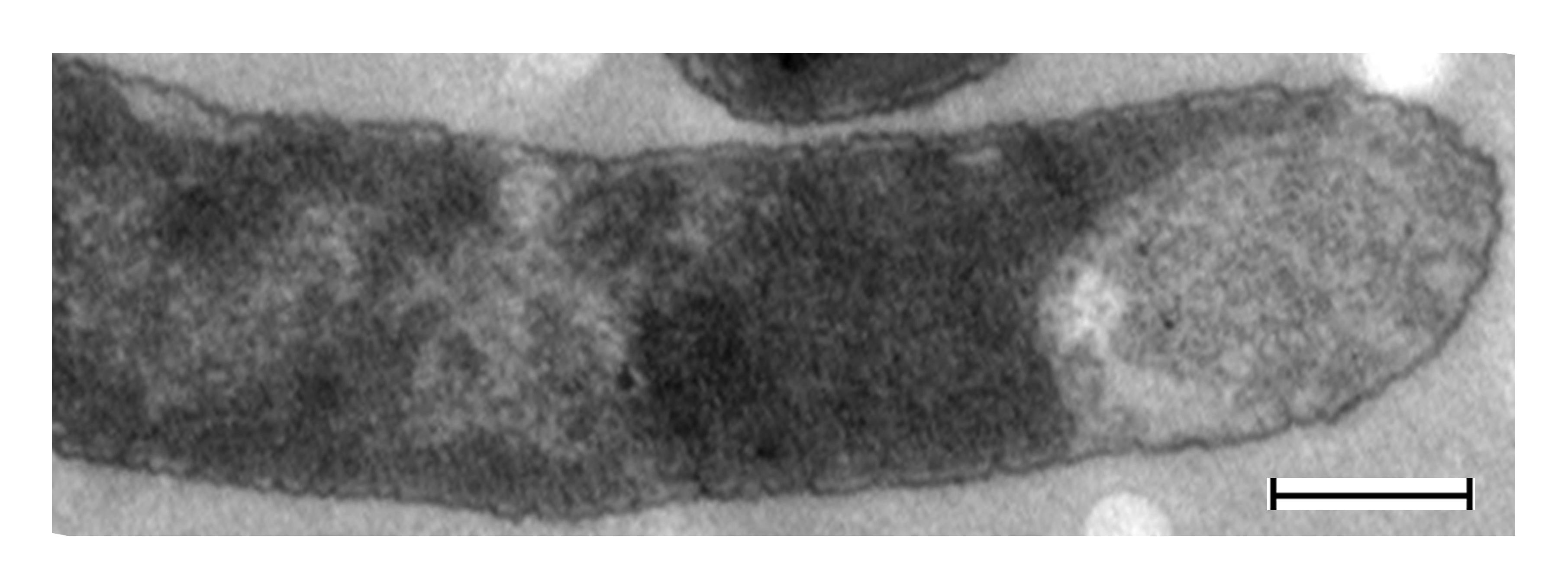


**Figure S1. Transmission electron microscopy images of a formazan granule in a filamentous cell of *E. coli* strain UT481.** Scale bar equals 400 nm.
