# Supplementary material for: Vital Dye Reaction and Granule Localization in Periplasm of Escherichia coli
Source: PLoS One. 2012 Jun 4;7(6):e38427. doi: 10.1371/journal.pone.0038427 (PMC3366950; doi:10.1371/journal.pone.0038427)
Supplement: Figure S2 — Schematic of cell geometry used for simulation. (DOC) [file pone.0038427.s002.doc]

**Figure S2. Schematic of cell geometry used for simulation.** Inner membrane is a shell with radius R_I_ and outer membrane is a shell with radius R_O_. The total length of the cell is *L* + 2*R_O_* and the width of the cytoplasmic space is *R_O_* – *R_I_*. The aspect ratio (*L* + *2R_O_*)/*2R_O_* is chosen to be two. Aggregate is shown in green at two different cell locations, midcell and the left-hand pole.
