# Supplementary material for: Vital Dye Reaction and Granule Localization in Periplasm of Escherichia coli
Source: PLoS One. 2012 Jun 4;7(6):e38427. doi: 10.1371/journal.pone.0038427 (PMC3366950; doi:10.1371/journal.pone.0038427)
Supplement: Figure S3 — Heat map of aggregate sizes corresponding to different periplamic locations (horizontal axis) and different numbers of particles per cell (vertical axis). (DOC) [file pone.0038427.s003.doc]

**Figure S3. Heat map of aggregate sizes corresponding to different periplamic locations (horizontal axis) and different numbers of particles per cell (vertical axis).** (a) The influence of the thickness of the periplasmic space. The thickness of the periplasm was changed by varying the radius of the inner surface: i) R_I_ = 1.5σ, ii) R_I_=2σ, iii) R_I_ = 2.5σ, and iv) R_I_ = 3σ. (b) The influence of the interaction energy *ε* between particles. The interaction energy ε was varied from i) 1.75k_B_T, ii) 2k_B_T, and 2.25/k_B_T. In all simulations the rate of adding molecules was N_add_ = 50000 MC steps.
