# Supplementary material for: Vital Dye Reaction and Granule Localization in Periplasm of Escherichia coli
Source: PLoS One. 2012 Jun 4;7(6):e38427. doi: 10.1371/journal.pone.0038427 (PMC3366950; doi:10.1371/journal.pone.0038427)
Supplement: Figure S4 — Estimation of the growth rate of formazan granules in E. coli periplasm. (DOC) [file pone.0038427.s004.doc]

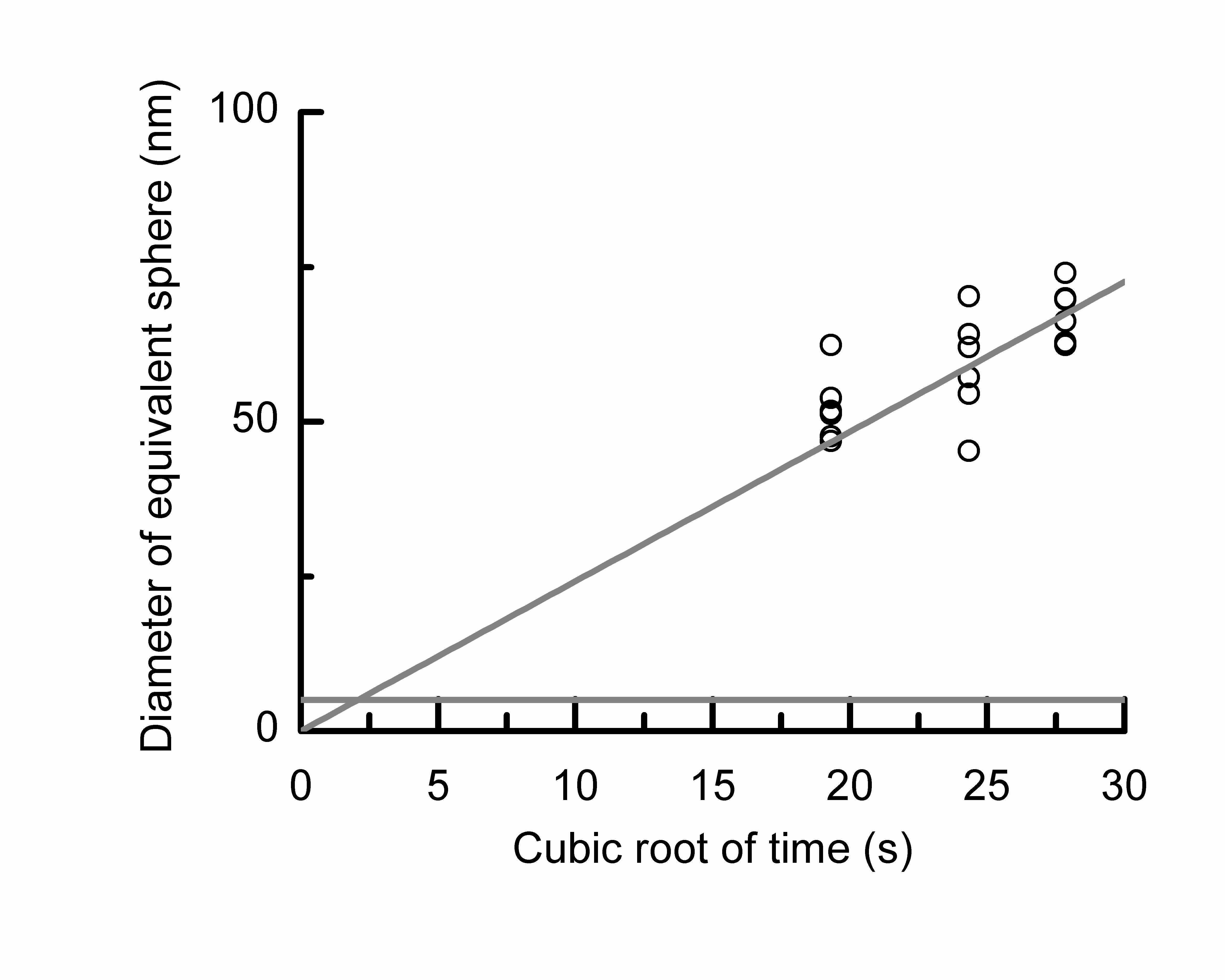


**Figure S4. Estimation of the growth rate of formazan granules in *E. coli* periplasm.** The *E. coli* cells cultivated for 2 h, 3 h, and 4 h in presence of 0.005% TTC were fixed and ultrathin sectioned. Six largest cutting surfaced of granules were selected. They presumably reflected the areas of the mid-plane of the formazan granules, because no obvious membrane deformation has been observed up to 12 hours of incubation. The horizontal line is at 5 µm. A spherical granule of this size would be trapped in the periplasm. We estimate the volume of formazan molecules as ~0.28 nm^3^ according to the parameters published in [1], and the reduction rate was calculated as 170 molecules per second, i.e. the cell reduced 1 molecule very 6 ms. The extrapolation of the above curve indicates that a seed need ~10 s to grow large enough to be trapped in the periplasm. Such a granule would contain 1853 formazan molecules.

**REFERENCES**

1. Pauling L (1960) The nature of chemical bond. New York: Cornell University Press.
