# Supplementary material for: Vital Dye Reaction and Granule Localization in Periplasm of Escherichia coli
Source: PLoS One. 2012 Jun 4;7(6):e38427. doi: 10.1371/journal.pone.0038427 (PMC3366950; doi:10.1371/journal.pone.0038427)
Supplement: Figure S5 — The localization of formazan granules in cells of strain AW405 growing in medium supplemented with different amount of TTC. (DOC) [file pone.0038427.s005.doc]

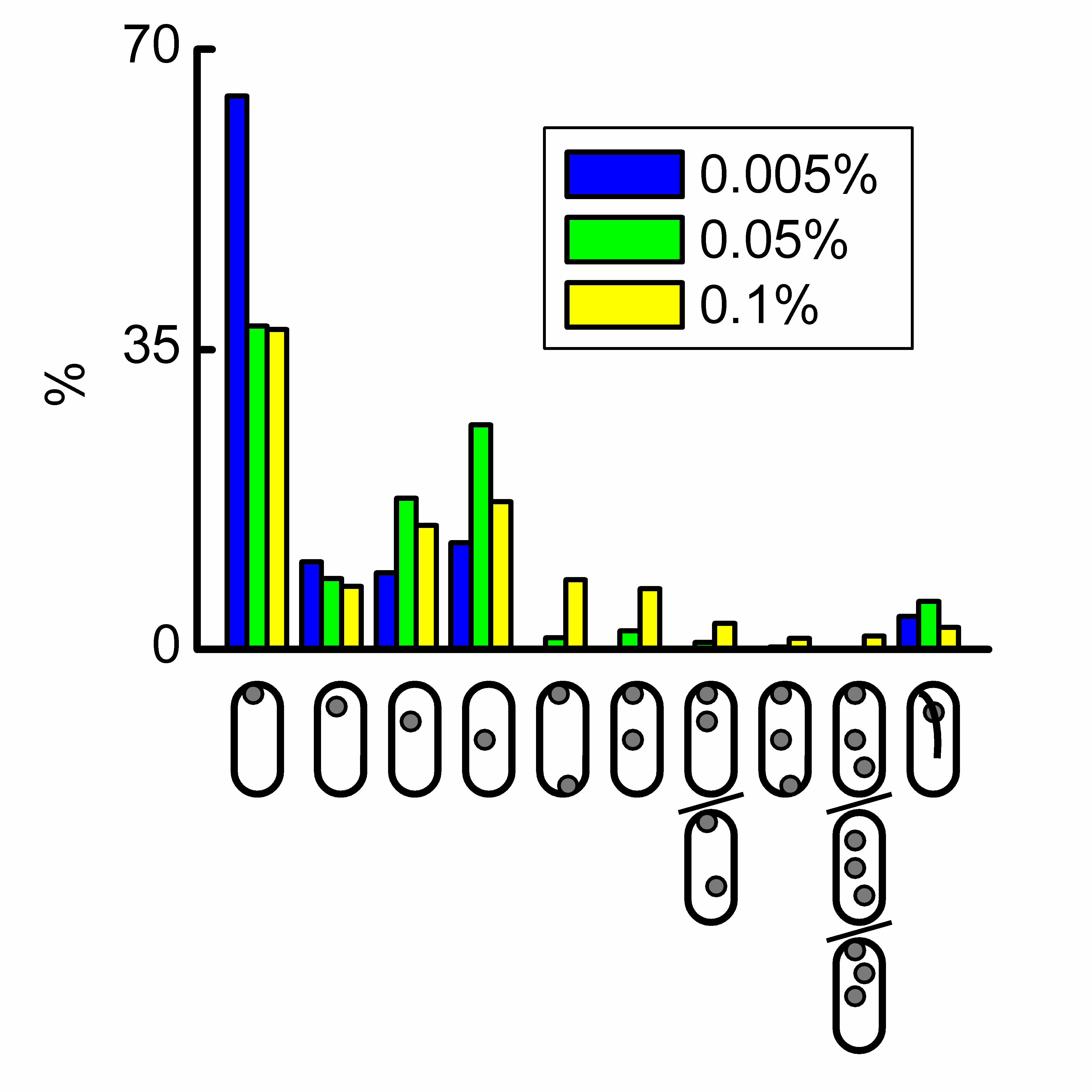


**Figure S5. The localization of formazan granules in cells of strain AW405 growing in medium supplemented with different amount of TTC.** Diagrams of cells with differently localized granules were shown on the x-axis. Note that both the first and second types were localized at the pole. The cells of strain AW405 is slight larger than those of strain LMG194, thus allow distinguishing between cells with granules in the pole cape and cells with granules near the pole base. The very last diagraph represents cells with a formazan strip.
