# Supplementary material for: Vital Dye Reaction and Granule Localization in Periplasm of Escherichia coli
Source: PLoS One. 2012 Jun 4;7(6):e38427. doi: 10.1371/journal.pone.0038427 (PMC3366950; doi:10.1371/journal.pone.0038427)
Supplement: Table S1 — Bacterial strains and their TTC reduction activities. (DOC) [file pone.0038427.s006.doc]

**Table S1. Bacterial strains and their TTC reduction activities**.

| ***E. coli*** **strain** | **Genotype** | **Growth condition** | **TTC Reduction** | **Source** |
| --- | --- | --- | --- | --- |
| MC1000 | *ara*D139, *Δ*(*ara*, *leu*)7697, *Δlac*X74, *gal*U, *gal*K, *str*A | **Aerobic** | **Yes** | [1] |
| MC1000*ΔdsbA* | *ara*D139, *Δ*(*ara*, *leu*)7697, *Δlac*X74, *gal*U, *gal*K, *str*A, *ΔdsbA*::kan | **Aerobic** | **Extensive** | Lab stock |
| MC1000*ΔdsbD* | *ara*D139, *Δ*(*ara*, *leu*)7697, *Δlac*X74, *gal*U, *gal*K, *str*A, *ΔdsbD* | **Aerobic** | **Yes** | [2] |
| MC1000*ΔdsbA/ΔdsbD* | *ara*D139, *Δ*(*ara*, *leu*)7697, *Δlac*X74, *gal*U, *gal*K, *str*A, *ΔdsbA*::kan, *ΔdsbD* | **Aerobic** | **Yes** | Lab stock |
| MC1061 | *ara*D139, *Δ*(*ara*, *leu*)7697, *Δlac*X74, *gal*U^-^, *gal*K^-^*hsr*^-^, *hsm*^+^, *str*A | **Aerobic** | **Yes** | [1] |
| EC06 | *∆ccmA-H* F^-^ *hsdR mcrB ara-D139 ∆(araABC-leu)7679 galU gaK ∆(lac)X74 rpsL thi* | **Aerobic** | **Yes** | [3] |
| AN387 | *F- rpsL gal* | **Aerobic**  **Anaerobic** | **Yes**  **Yes** | [4] |
| AN386 | *F- rpsL gal menA401* | **Aerobic** | **Yes** | [4] |
| AN385 | *F- rpsL gal ubiA420* | **Anaerobic** | **Yes** | [4] |
| AN384 | *F- rpsL gal ubiA420 menA401* | **Anaerobic** | **No** | [4] |

**REFERENCES**

1. Casadaban MJ, Cohen SN (1980) Analysis of gene control signals by DNA fusion and cloning in Escherichia coli. J Mol Biol 138: 179-207.

2. Stewart EJ, Katzen F, Beckwith J (1999) Six conserved cysteines of the membrane protein DsbD are required for the transfer of electrons from the cytoplasm to the periplasm of Escherichia coli. EMBO J 18: 5963-5971.

3. Thöny-Meyer L, Fischer F, Kunzler P, Ritz D, Hennecke H (1995) Escherichia coli genes required for cytochrome c maturation. J Bacteriol 177: 4321-4326.

4. Imlay JA (1995) A metabolic enzyme that rapidly produces superoxide, fumarate reductase of Escherichia coli. J Biol Chem 270: 19767-19777.
